# Phylogenetic analysis revealed the co-circulation of four dengue virus serotypes in Southern Thailand

**DOI:** 10.1371/journal.pone.0221179

**Published:** 2019-08-15

**Authors:** Rodolphe Hamel, Pornapat Surasombatpattana, Sineewanlaya Wichit, Alexandra Dauvé, Celeste Donato, Julien Pompon, Dhanasekaran Vijaykrishna, Florian Liegeois, Ronald Morales Vargas, Natthanej Luplertlop, Dorothée Missé

**Affiliations:** 1 MIVEGEC UMR 224, Université de Montpellier, IRD, CNRS, Montpellier, France; 2 Department of Microbiology and Immunology, Faculty of Tropical Medicine, Mahidol University, Bangkok, Thailand; 3 Pathology Department, Prince of Songkla University, Hat Yai, Thailand; 4 Faculty of Medical Technology, Mahidol University, Bangkok, Thailand; 5 Department of Entomology, Faculty of Tropical Medicine, Mahidol University, Bangkok, Thailand; 6 Department of Microbiology, Biomedicine Discovery Institute, Monash University, Clayton, Australia; 7 Programme in Emerging Infectious Diseases, Duke-NUS Medical School, Singapore, Singapore; CEA, FRANCE

## Abstract

Dengue fever is caused by dengue viruses (DENV) from the *Flavivirus* genus and is the most prevalent arboviral disease. DENV exists in four immunogenically distinct and genetically-related serotypes (DENV-1 to 4), each subdivided in genotypes. Despite the endemicity of all four DENV serotypes in Thailand, no prior study has characterized the circulation of DENV in the southern provinces of the country. To determine the genetic diversity of DENV circulating in Southern Thailand in 2015 and 2016, we investigated 46 viruses from 182 patients’ sera confirmed positive for DENV by serological and Nested RT-PCR tests. Our dataset included 2 DENV-1, 20 DENV-2, 9 DENV-3 and 15 DENV-4. Phylogenetic analysis was performed on viral envelop sequences. This revealed that part of the identified genotypes from DENV-1 and DENV-4 had been predominant in Asia (genotype I for both serotypes), while genotype II for DENV-4 and the Cosmopolitan genotype DENV-2 were also circulating. Whereas DENV-3 genotype II had been predominantly detected in South East Asia during the previous decades, we found genotype III and genotype I in Southern Thailand. All DENV genotype identified in this study were closely related to contemporary strains circulating in Southeast Asian countries, emphasizing the regional circulation of DENV. These results provide new insights into the co-circulation of all four DENV serotypes in Southern Thailand, confirming the hyperendemicity of DENV in the region. These findings also suggest a new trend of dissemination for some DENV serotypes with a possible shift in genotype distribution; as recently observed in other Asian countries.

## Introduction

Dengue viruses (DENV) have become the most prevalent arboviruses in the world [1]. DENV pose a significant public health and economic burden worldwide with more than 2.5 billion people living in regions of high-risk of infection, particularly in high population density urban centers in inter-tropical countries [2]. Symptoms range from fever, characterized by a flu-like febrile illness with or without warning signs, to severe dengue with plasma leakage, bleeding or organ impairment [3]. Despite the availability of a licensed vaccine against DENV in several countries, the poor efficacy of the vaccine has prevented a global vaccination program. Additionally, there are no specific antiviral treatment against DENV infection [4].

DENV belong to the genus *Flavivirus* of the *Flaviviridae* family. There are four immunogenically and genetically distinct DENV serotypes (DENV-1 to 4). Each of the DENV serotypes are further classified into 3–5 genotypes based on genetic divergence [5]. While all four serotypes are commonly observed in DENV endemic regions around the world, genotypes within each of the DENV serotypes are usually separated geographically.

In Thailand, all four DENV serotypes are endemic and more than 80,000 cases were reported in 2014 [6]. The first cases of dengue in the Kingdom of Thailand were reported in 1949 [7]. Major outbreaks of Dengue have been reported since the 1960’s associated with different serotypes. Dengue continues to cause regular epidemics and still remains a public health priority for Thailand’s authorities [8]. The southern region of Thailand is located in the heart of Southeast Asia at the crossroads of several countries where dengue is endemic. The entanglement of rural and urban territories and the movement of inhabitants are conducive to the maintenance of DENV and to the emergence of outbreaks in the human community.

Previous studies showed that all DENVs circulated continuously in Thailand with different trends for each serotype [9, 10]. This situation is not unique as DENV serotypes are endemic in many countries, resulting in so called hyperendemicity [11–16]. However, even though it was proposed that the hyperendemicity could aggravate the frequency of dengue severe symptoms, the relationship between concurrent infection and disease severity remain contradictory, with variable conclusions depending on the studies [17]. The co-circulation and the replacement of genotype are also associated with virulence and have already been reported [18–21]. In Thailand, genotype replacement for DENV-2 and DENV-3 was reported during the 1980’s and the 1990’s, respectively [22, 23].

Despite the presence of DENV in the four regions of Thailand, Limkittikul *et al* showed the heterogeneity of the geographical patterns of dengue desease from 2000 to 2011 [9]. This heterogeneity included serotype distribution variation in time and place, but also a periodic higher incidence rate reported in the south of Thailand compared to the other 3 regions. Southern Thailand is an important area for trade and tourism. The southern provinces of Narathiwat, Satun, Songkhla and Yala are land and air gateways into Thailand and are involved in the North-South terrestrial communication between continental Asian countries on the one hand and the large insular region of Southeast Asia (SEA) on the other hand.

Phylogenetic analysis of DENV sequences collected through surveillance allows monitoring of the spatiotemporal dissemination of DENV, which can show the potential rise of a specific genotype in an area or highlight a shift of genotype, as well as changes in virulence, vector competence, geographical adaptation and major epidemic episodes as previously reported [20, 24, 25]. In a DENV-ridden country, monitoring the circulation of the different strains should provide a better understanding of the dynamic of the virus and give clues on the impact of specific serotype/genotype in local epidemics.

Here we used a phylogenetic approach to investigate DENV circulation in the southern provinces of Thailand during 2015–2016 and determine the origins and routes of introduction of the DENV strains. While previous studies focused on a single serotype [26, 27] or were restricted in certain areas in Thailand [28, 29], our objective is to explore, for the first time, the local genetic diversity of the four DENV serotypes and determine whether new strains are emerging or if a switch of genotype is ongoing in Southern Thailand.

## Material and methods

### Study area, sample collection and characterization

**Fig 1:**
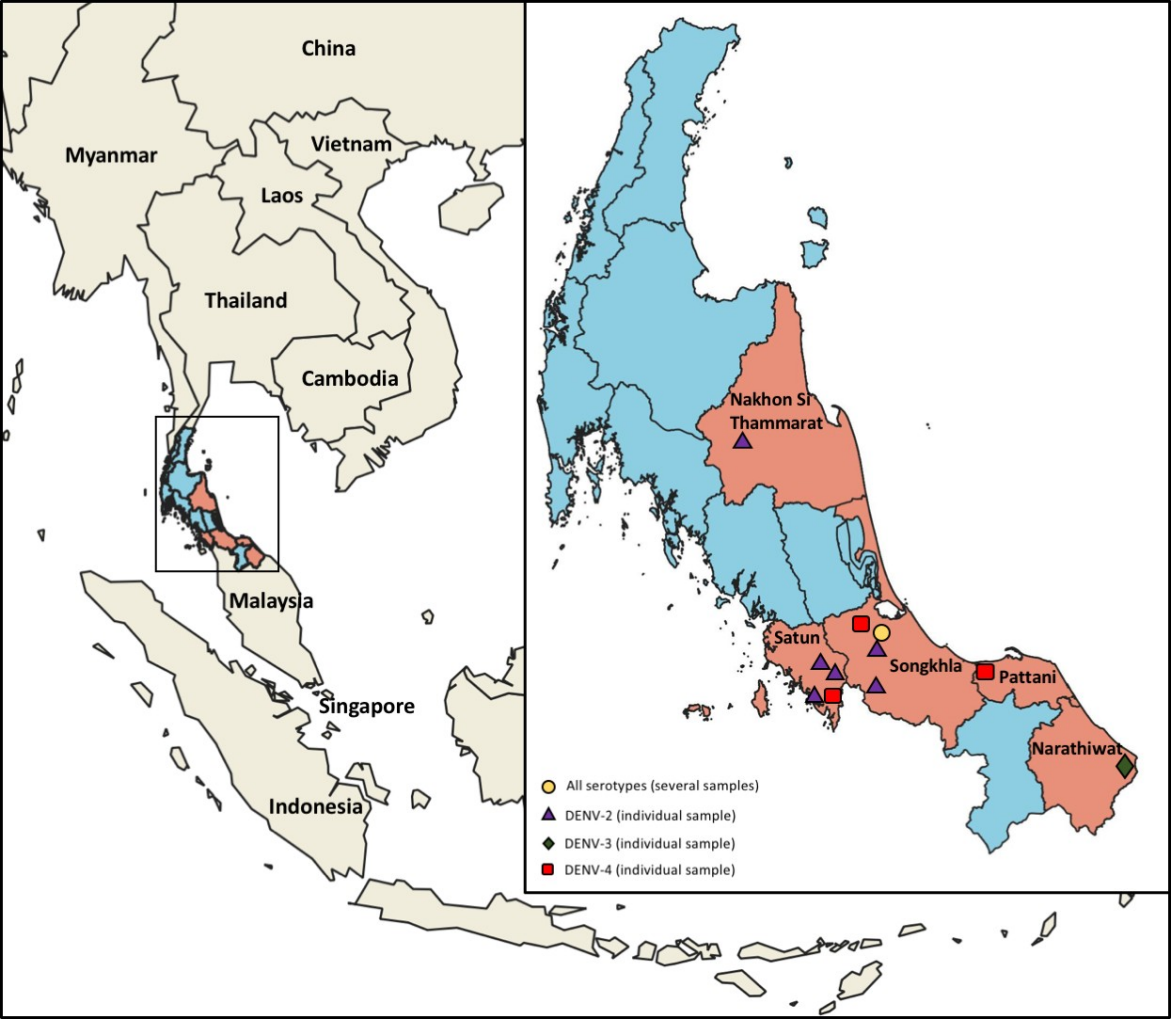
Map of Thailand showing surveillance region and DENV types detected. Participating provinces are highlighted in orange. Location from which the patients originated are marked on the map along with DENV serotype: Purple triangles, DENV-2; green diamond, DENV-3; red square, DENV-4; and yellow circle multiple serotypes. The map was annotated using QGIS software.

Serum samples were collected from 182 dengue patients in the acute-phase of infection originated from five provinces in Southern Thailand; namely Nakhon Si Thammarat, Narathiwat, Pattani, Satun and Songkhla (Fig 1 and Supplementary S1 Table). Only patients exhibiting febrile illness (temperature >38°C), and who were suspected of arbovirus infection, according to the World Health Organization (WHO) guideline for dengue diagnosis were included in the study [30]. Serological testing was performed on patient blood samples using a rapid DENV non-structural protein 1 (NS1) antibody test (SD BIOLINE Dengue Duo rapid test; Abbot Laboratories, Bangkok, Thailand). Description of study population is recorded in S1 Table.

### RT-PCR amplification, serotyping and DENV envelope gene sequencing

Serotyping was conducted on all NS1 positive samples using a nested RT-PCR as previously described [31]. Viral RNA was extracted from all NS1 positive samples using the QIAamp Viral RNA mini kit (Qiagen, Bangkok, Thailand). 140μL of serum samples were used for extraction following the manufacturer’s protocol. Purified viral RNAs were stored at -80°C until the serotyping and sequencing was performed. Nested RT-PCR was performed using the SensiFast kit (Bioline, Menphis, TN, USA). The first PCR was performed using the primer pair D1F and D1R with the following cycling conditions: activation at 95°C for 10 min followed by 35 amplification cycles of 95°C for 5s, 57°C for 20s and 72°C for 1min. The nested PCR was performed on the first PCR amplicon using forward primer D1F and serotype specific reverse primer (RTS1, RTS2, RTS3 and RTS4) with the following program: activation at 95°C for 10min followed by 30 amplification cycles of 95°C for 15s, 58°C for 20min and 72°C for 1min. PCR products were visualized on a 1.8% agarose gel. Primers used to determine the serotype of the virus are provided in S2 Table.

To avoid working on viruses passaged and isolated from cell culture, we decided to directly sequence samples from viral RNA previously extracted from serum as mentioned above. Then, isolated RNA were retro-transcribed in cDNA using random hexamer and MMLV reverse transcription kit (Promega, Charbonnière, France), following the manufacturer’s protocol. Then, PCR was performed to amplify the full Envelope (Env) gene sequence using KAPA2G Fast Ready Mix (KapaBiosystem, Wilmington, MA, USA) following manufacturer’s instructions. The 1^st^-forward and 4^th^-reverse primers for each serotype were used to amplify Env gene sequences (S2 Table). Amplification was performed using the following cycling conditions: activation at 95°C for 10 min followed by 40 amplification cycles of 95°C for 15s, 60°C for 15s and 72°C for 30s. PCR products were visualized on a 1.5% agarose gel and specific amplicons were purified from gel using QIAquick PCR Purification kit (QIAGEN, Bangkok, Thailand) following manufacturer’s instructions and stored at -20°C until sequencing was performed. The entire envelope gene was successfully amplified for 46 samples. Purified amplicons were directly sequenced in both 5’ and 3’ directions using cycle sequencing and dye terminator methodologies by Macrogen company (Seoul, Korean). A set of eight overlapping primers for each serotype were used for sequencing (S2 Table) in order to cover all the entire gene and avoid discrepancies.

### Phylogenetic analysis

To characterize the DENV isolated from clinical cases in Southern Thailand, the sequences encoding the envelope protein E were subject to robust phylogenetic analyses, along with representative DENV sequences obtained from the NCBI GenBank database (S3 Table). Sequences were selected to cover a broad geographical area and includes sequences from countries where DENV circulation were reported from several decades. All sequences were referenced into the tree in a format consisting of “accession number_country_year of isolation”.

Overlapping reads were assembled into contiguous sequences using CAP3 Sequence Assembly Program [32]. Multiple sequence alignments were conducted using MEGA 7 [33]. Sequences were edited and sites that could not be unambiguously aligned were excluded from subsequent analyses. Maximum likelihood (ML) trees were constructed using PhyML software [34] with the best-fit nucleotide substitution model (GTR+G)identified by Akaike's Information criterion (AIC) using Topali v.2 [35]. Bootstrap method was used to measure the robustness of nodes with 1000 iterations. Phylogenetic trees were edited with FigTree v1.4.3 software (http://tree.bio.ed.ac.uk/software/figtree/). DENV serotypes 1, 2, 3 and 4 were classified following Goncalvez *et al*, Twiddy *et al*, Lanciotti *et al* and Lanciotti *et al*, respectively [36–39]. All sequences from our study have been deposited in GenBank database and their accession numbers are shown in Table 1.

**Table 1:** Information on dengue-infected patients from whom DENV E protein gene were sequenced.

Code of samples	Age*	Sex**	Location (Town/Province)	Date of collection (month/year)	Serotype	Genotype	GenBank accession number
DC201	8	F	HatYai/Songkla	Nov 2016	1	I	MK780858
DC202	19	M	HatYai/Songkla	Nov 2016	1	I	MK780859
DC9	27	F	Khuan Don/Songkhla	Sep 2015	2	Cosmopolitan	MK780860
DC21	5	M	HatYai/ Songkhla	Oct 2015	2	Cosmopolitan	MK780861
DC30	24	M	HatYai/ Songkhla	Oct 2015	2	Cosmopolitan	MK780862
DC35	10	F	Na Mon/Songkhla	Nov 2015	2	Cosmopolitan	MK780863
DC39	30	F	Khuan Kalong/Songkhla	Nov 2015	2	Cosmopolitan	MK780864
DC40	12	M	HatYai/ Songkhla	Nov 2015	2	Cosmopolitan	MK780865
DC58	12	F	Satun/Satun	Apr 2016	2	Cosmopolitan	MK780866
DC61	11	F	Khlong Hoi Khong/ Songkhla	May 2016	2	Cosmopolitan	MK780867
DC66	28	M	HatYai/ Songkhla	Jun 2016	2	Cosmopolitan	MK780868
DC70	20	F	HatYai/ Songkhla	Aug 2016	2	Cosmopolitan	MK780869
DC4	19	F	HatYai/ Songkhla	Sep 2015	2	Asian I	MK780870
DC11	20	M	HatYai/ Songkhla	Sep 2015	2	Asian I	MK780871
DC13	48	M	HatYai/ Songkhla	Sep 2015	2	Asian I	MK780872
DC14	35	F	HatYai/ Songkhla	Dec 2015	2	Asian I	MK780873
DC16	16	M	HatYai/ Songkhla	Jan 2016	2	Asian I	MK780874
DC23	25	F	Pakpayoon/Songkhla	Oct 2015	2	Asian I	MK780875
DC26	33	F	HatYai/ Songkhla	Oct 2015	2	Asian I	MK780876
DC49	27	M	Tung Song/ Nakhon Si Thammarat	Feb 2016	2	Asian I	MK780877
DC57	13	M	Sadao/ Songkhla	Apr 2016	2	Asian I	MK780878
DC65	22	F	HatYai/ Songkhla	Jun 2016	2	Asian I	MK780879
DC12	54	F	HatYai/ Songkhla	Sep 2015	3	I	MK780880
DC32	20	F	HatYai/ Songkhla	Oct 2015	3	I	MK780881
DC50	29	M	Takbai/ Narathiwat	Feb 2016	3	I	MK780882
DC1	13	M	HatYai/ Songkhla	Sep 2015	3	III	MK780883
DC2	47	M	Takbai/ Narathiwat	Sep 2015	3	III	MK780884
DC3	21	F	HatYai/ Songkhla	Sep 2015	3	III	MK780885
DC6	13	F	HatYai/ Songkhla	Sep 2015	3	III	MK780886
DC24	11	M	HatYai/ Songkhla	Sep 2015	3	III	MK780887
DC125	29	M	HatYai/ Songkhla	Nov 2016	3	III	MK780888
DC38	20	F	HatYai/ Songkhla	Nov 2015	4	I	MK780889
DC44	20	F	HatYai/ Songkhla	Dec 2015	4	I	MK780890
DC46	30	F	HatYai/ Songkhla	Jan 2016	4	I	MK780891
DC54	24	M	HatYai/ Songkhla	Mar 2016	4	I	MK780892
DC56	7	F	HatYai/ Songkhla	Mar 2016	4	I	MK780893
DC67	62	M	Khok Pho/ Pattani	Jun 2016	4	I	MK780894
DC85	13	F	HatYai/ Songkhla	Sep 2016	4	I	MK780895
DC100	15	F	HatYai/ Songkhla	Oct 2016	4	I	MK780896
DC113	9	F	Satun/ Satun	Oct 2016	4	I	MK780897
DC19	23	M	HatYai/ Songkhla	Oct 2015	4	II	MK780898
DC28	15	M	HatYai/ Songkhla	Oct 2015	4	II	MK780899
DC33	2	F	HatYai/ Songkhla	Nov 2015	4	II	MK780900
DC36	28	F	HatYai/ Songkhla	Nov 2015	4	II	MK780901
DC41	28	M	HatYai/ Songkhla	Nov 2015	4	II	MK780902
DC42	19	F	Rattaphum/Songkhla	Nov 2015	4	II	MK780903

Origins of dengue strains used in the study

*Age of patients in years.

**F: female, M: male

### Ethics statement

This study was approved by the ethical committee and the samples were carried out following the rules and laws that govern the use of human material in Thailand. The study protocol was approved by the Ethical Committees of the Faculty of Tropical Medicine, Mahidol University (Bangkok, Thailand) (approval No. MUTM 2016-002-01), the Faculty of Medicine of Prince of Songkhla University and Hat Yai Hospital (Songkhla, Thailand) (approval No. 59-159-05-2). Informed consent was obtained from study participants and for minors from parents or a legal representative.

## Results

Of the 182 serum samples collected in the southern province of Thailand during 2015–2016, from patients presenting with dengue-like symptoms and confirmed positive for dengue by both NS1 serological test and RT-PCR, the envelope gene of 46 were successfully sequenced. The serotype was determined by nested RT-PCR, revealing the co-circulation of all four DENV serotypes in Southern Thailand (Table 1; DENV1, 2; DENV2, 20; DENV3, 9; DENV4, 15). Overall, we detected the four serotypes in overlapping and distinct regions, revealing a complex hyperendemicity dynamic in the region (Fig 1).

**Fig 2:**
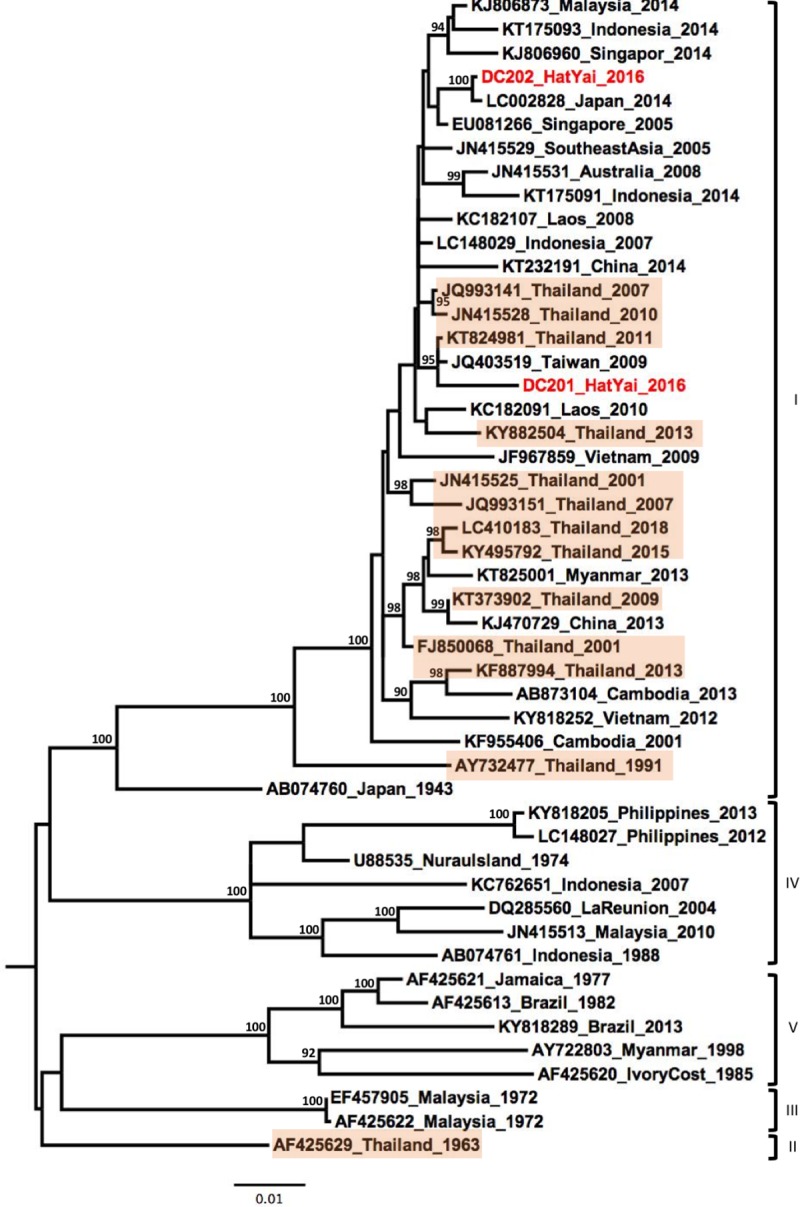
Phylogenetic analysis of DENV-1. Maximum-likelihood tree of dengue virus serotype 1 envelope gene sequences, generated using the GTR+G substitution model. Bootstrap values are shown on branch nodes. Samples collected in this study are in red colour font and strains from Thailand are highlighted in light orange. Annotation on the right denote DENV genotype.

Phylogenetic analysis of the two DENV-1 samples showed their affiliations to the genotype I and that they grouped with contemporary strains isolated in Southeast Asia between 2005–2014 (Fig 2).

**Fig 3:**
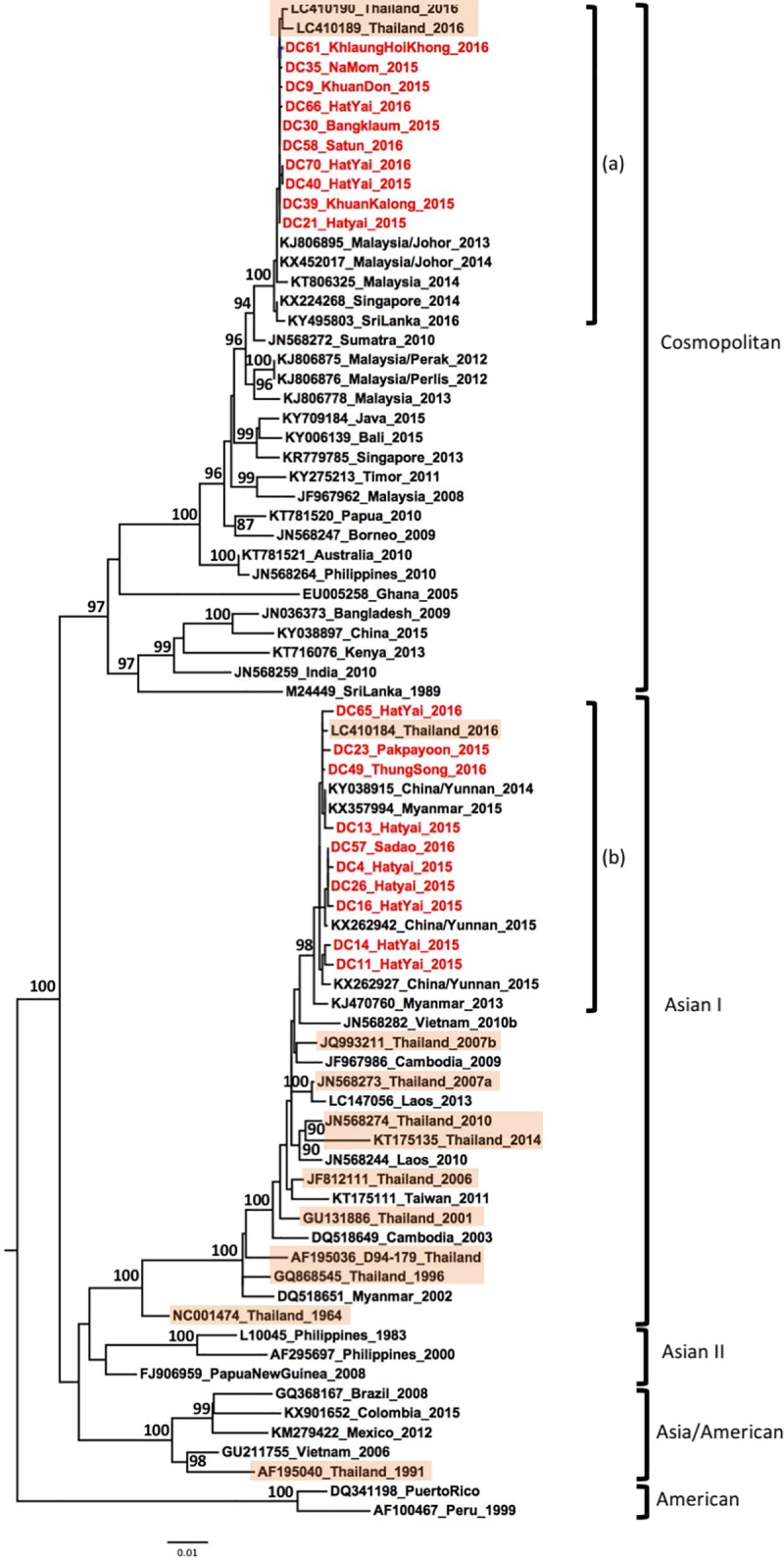
Phylogenetic analysis of DENV-2. Maximum-likelihood tree of dengue virus serotype 2 envelope gene sequences, generated using the GTR+G substitution model. Bootstrap values are shown on branch nodes. Samples collected in this study are in red colour font and strains from Thailand are highlighted in light orange. Annotation on the right denote DENV genotype.

Of the twenty DENV-2 samples, ten belonged to the Cosmopolitan genotype. These formed a monophyletic lineage that was closely related to strains isolated in Malaysia, Singapore, Sri Lanka since 2013, as well as strains isolated from north and central parts of Thailand in 2016 [29], suggesting endemicity of the virus in the region (Fig 3, label a). The remaining ten strains belonged to the genotype Asian I and formed a monophyletic lineage comprising strains isolated in the province of Yunnan, China, in 2015 [40], in Myanmar in 2015 [41] and central province in Thailand [29] (Fig 3, label b). The phylogenetic association of the Cosmopolitan genotypes with Southeast Asian countries and the Asian I genotype with China and Myanmar indicates spatial segregation of the DENV-2 genotypes within Asia, however both these genotypes overlapped in Southern Thailand.

**Fig 4:**
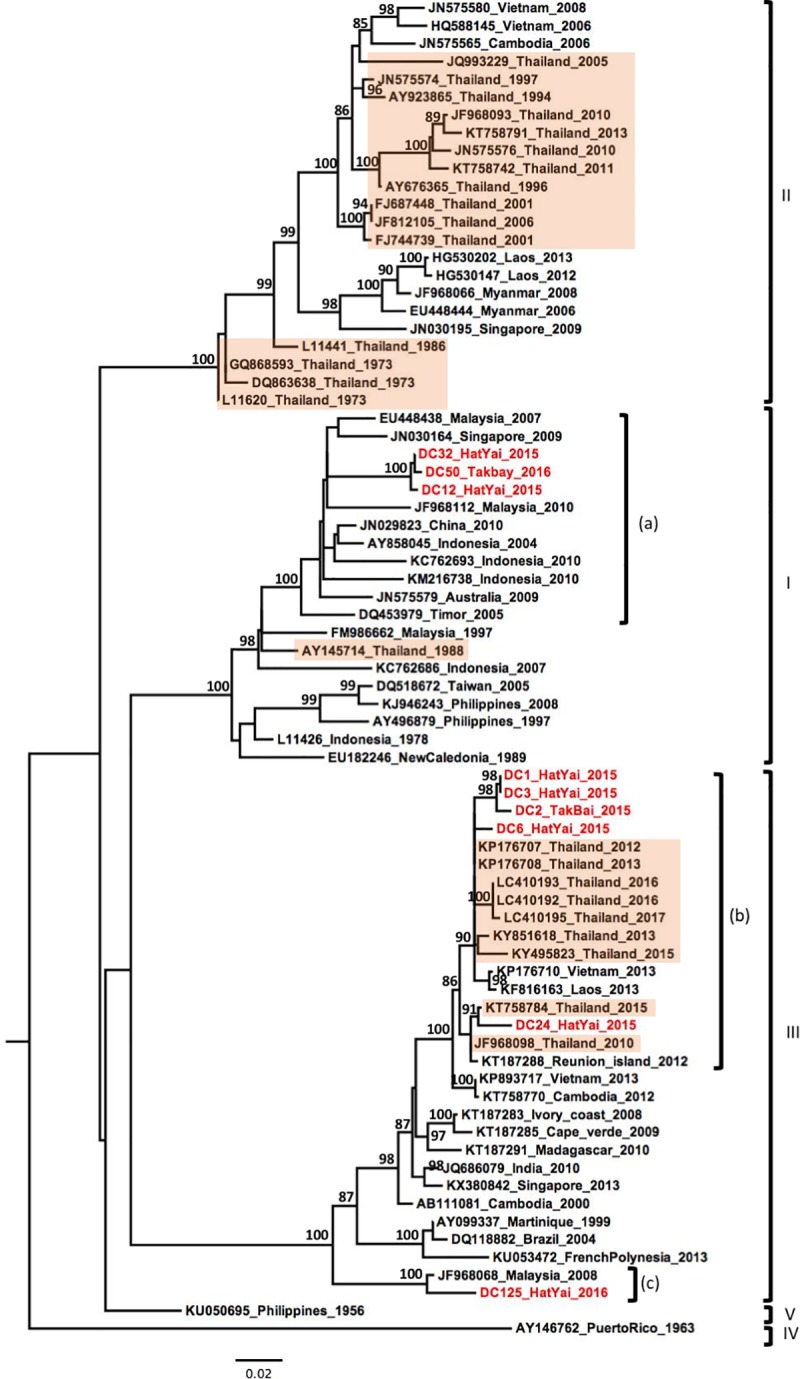
Phylogenetic analysis of DENV-3. Maximum-likelihood tree of dengue virus serotype 3 envelope gene sequences, generated using the GTR+G substitution model. Bootstrap values are shown on branch nodes. Samples collected in this study are in red colour font and strains from Thailand are highlighted in light orange. Annotation on the right denote DENV genotype.

Out of the nine DENV-3 samples, three were identified as genotype I (Fig 4). These grouped together and clustered with strains collected over the past decade from Southeast Asian countries (Indonesia, Singapore, Malaysia) and Australia (Fig 4, label a). The six remaining DENV-3 strains belonged to the genotype III, although they were derived from multiple lineages circulating over the last decade in Southeast Asian countries, including Laos, Vietnam, Malaysia and Thailand (Fig 4, labels b and c).

**Fig 5:**
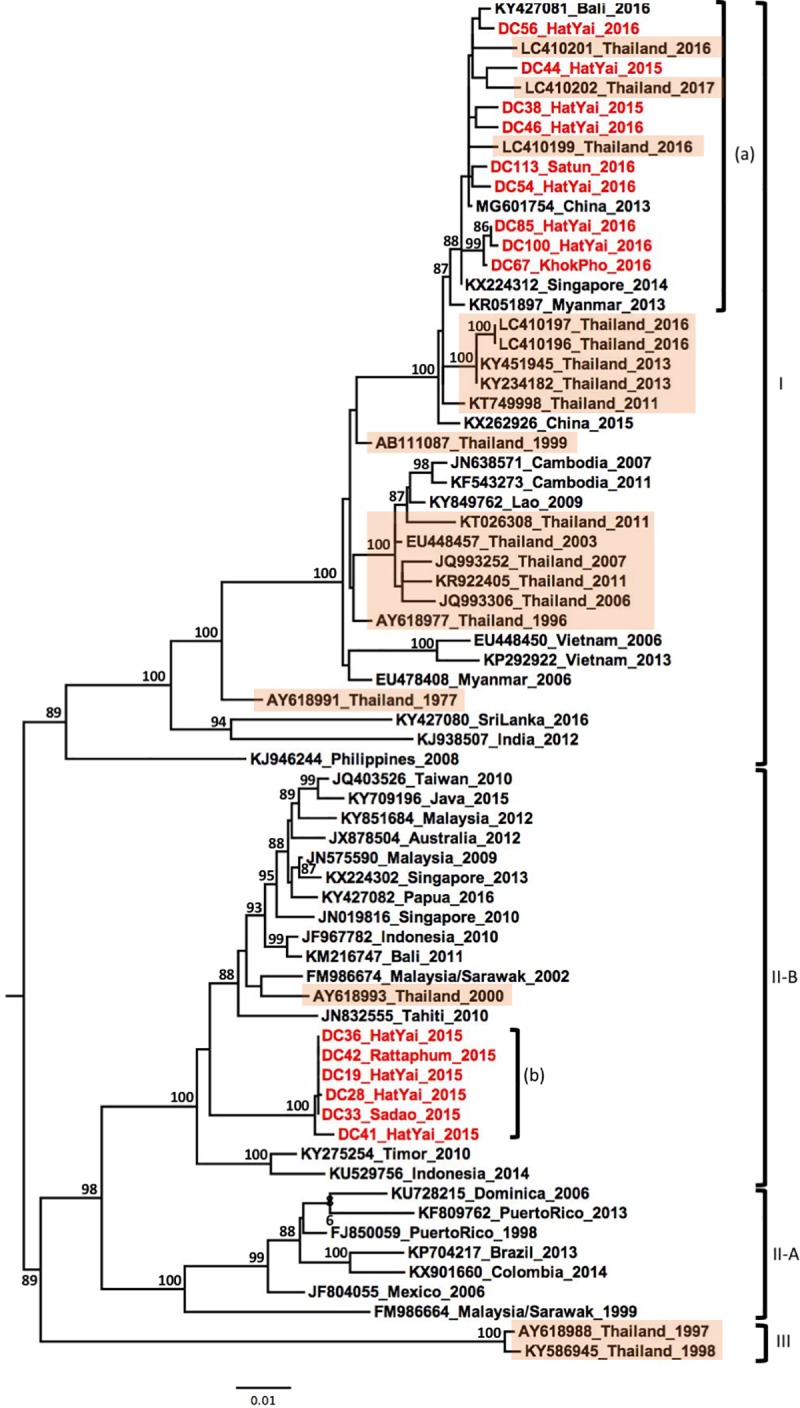
Phylogenetic analysis of DENV-4. Maximum-likelihood tree of dengue virus serotype 1 envelope gene sequences, generated using the GTR+G substitution model. Bootstrap values are shown on branch nodes. Samples collected in this study are in red colour font and strains from Thailand are highlighted in light orange. Annotation on the right denote DENV genotype.

Similar to other serotypes, DENV-4 samples collected from southern Thailand belonged to multiple genotypes. Nine samples belonged to the genotype I and clustered with strains collected in Central Thailand in 2016–2017, in Singapore in 2014 and in China in 2013, forming a monophyletic lineage (Fig 5, label a). The six remaining samples grouped in a well-supported lineage (Fig 5, label b) belonging to the genotype II, within clade II-B along with strains from Southeast Asia and Australia between 2000–2016 (Fig 5).

## Discussion

Screening of dengue samples collected from patients presenting with dengue-like symptoms to hospitals in southern Thailand during 2015–2016 identified all four DENV serotypes, highlighting the hyperendemicity in Southern Thailand. To determine the genotypes of DENV and the relationships with other DENV strains, particularly from Thailand and Southeast Asia, we generated sequences of DENV envelope gene directly from patient serum samples and conducted a comprehensive phylogenetic analysis.

DENV-1 viruses were identified as genotype I and showed a close genetic link with strains previously reported from several countries in Southeast Asia and the Pacific region. Recently, a DENV-1 strain (LC410183_Thailand_2016) was isolated from the province of Chang Mai in Northern Thailand [29]. Interestingly, the DENV-1 strains from our study were not grouped in the same clade with LC410183_Thailand_2016, suggesting intra-genotype diversity of genotype I strains circulating within Thailand.

Interestingly, DENV-2 samples belonged to two different genotypes. Ten samples belonged to the Asian I genotype, which has been commonly reported in several countries in Asia such as in Myanmar [42], Vietnam [43], Thailand [28, 29], Laos PDR [44] and Taiwan [45]. These 10 strains do not show a wide genetic diversity and cluster into a monophyletic lineage with DENV-2 strains isolated during a large outbreak in China in 2014 and 2015 (Province of Yunnan) and Myanmar 2015 [40, 41]. Furthermore, 10 other DENV-2 strains were classified as Cosmopolitan genotype and formed a specific clade into a single lineage with strains from Malaysia, Singapore, Sri Lanka as well as strains recently isolated from the Central and Northern region of Thailand. These strains were also related to viruses isolated from 2009 to 2016 in the insular region of Southeast Asia and Australia, where the Cosmopolitan genotype was the predominant genotype (Fig 3). As reported by Twiddy *et al*, the Cosmopolitan genotype is spreading worldwide, including throughout Southeast Asia [46]. Our analysis suggested that this genotype has also spread to Thailand, corroborating recent findings in the Central and Northern region of Thailand [29]. The Cosmopolitan genotype strains of our study are genetically close to strains from the Central and Northern regions of Thailand collected in 2016 [29]. Thus, our findings may suggest a route of introduction of DENV-2 into Thailand from the Malaysian peninsula or from other southern countries of Thailand, considering that our samples were collected in 2015 and 2016. Furthermore, these strains were genetically-related to the DENV-2 strains associated with outbreaks in Singapore and Malaysia in 2013 and 2014 [13, 47] (Fig 3). Further investigation is needed to assess whether several routes of introduction could be possible from countries to the north or south of Thailand. Both the genotype Asia I and Cosmopolitan genotype were reported to be regionally endemic [37, 48], suggesting that there is a frequent crossover between countries in Southeast Asia–highlighting the importance of continuously monitoring DENV in Southern Thailand. While the Asian/American genotype was displaced by the Asian-I genotype in Vietnam and Cambodia [20], our results suggest a shift towards the Cosmopolitan genotype in Thailand. However, to date, reports of the presence of the Cosmopolitan genotype are seldom [29, 49], highlighting the importance of permanent surveillance in Thailand.

In the past few years, several studies have shown the replacement of DENV-3 genotype II by the genotype III in Southeast Asia [18, 29]. Genotype III is now described as a widespread worldwide “Cosmopolitan genotype” with a high rate of dissemination upon introduction in a new area and has been linked to several outbreaks [18, 50]. Our phylogenetic analysis of six DENV-3 samples resulted in their classification within genotype III, suggesting that this genotype replacement occurred in Thailand between 2012–2013 (Fig 4). Our strains showed a genetic diversity with two distinct lineages classified within genotype III. Indeed, five samples were closely grouped in a monophyletic lineage with strains isolated from Thailand, Laos and Vietnam between 2012 to 2017, while the single strain DC-125 clustered separately with a strain isolated in Malaysia in 2008. This separation in two lineages suggests different routes of introduction or different modes of circulation in Thailand as proposed by Tan *et al* in Malaysia [18]. The strain DC125, closely related with the virus from Malaysia 2008, could have been introduced a few years ago and may have been circulating at a low level after its introduction in Southern Thailand. Furthermore, our observation in Southern Thailand corroborates the recent observations in the Central and Northern regions that also showed the establishment of genotype III [29]. Further investigation could confirm whether this is a general trend.

A significant result of our phylogenetic analysis of DENV-3 was that three samples were distributed into the genotype I. To the best of our knowledge, this is the first time since 1988 that the presence of genotype I has been reported in Thailand [23]. Our genotype I samples were grouped into a single clade closely related with viruses mostly circulating in Indonesia, Malaysia and Singapore, which were either isolated from travellers returning from this region [51, 52] or from natives during regional surveys and outbreaks [53, 54]. Our samples were collected from the district of HatYai in Songkhla province and the district of Takbai, in Narathiwat province, which shares a border with the Kelatan province in Malaysia. Previous studies reported a wide circulation of the genotype I in Malaysia and Indonesia [55]. These findings may suggest a spread of the DENV-3 genotype I from insular Southeast Asia into Southern Thailand and could lead to a replacement of the dominant genotype. However, future research is needed to determine whether genotype I is increasing in Thailand or whether our finding resulted from a time specific event in which genotype I was circulating in the region of our study.

Following the same pattern as the DENV-3 strains, the DENV-4 strains were grouped into two genotypes: lineage (a) which groups nine samples in genotype I and the lineage (b) which groups six strains in genotype II clade B (Fig 5). This observation shows that lineage (a) is closely related with strains isolated during outbreaks in Singapore in 2014 [13], in China from an imported case from Thailand (2013), Bali (2016) [56] and also recently isolated strains from Northern Thailand [29]. Genotype I is commonly found in Thailand and continental countries of Southeast Asia. However, the observation of lineage (b) suggests a possible an introduction that did spread and not established in introduction of a new genotype into Thailand. Indeed, six samples were grouped together into a single lineage belonging to genotype II. This clade formed a monophyletic lineage related to the Southeast Asian and Australian strains. The last detection of this genotype in Thailand was reported in 2000 in Bangkok [27]. It should be noted that all these samples were collected in 2015 from the province of Songkhla and had a low phylogenetic diversity. This observation may suggest a singular introduction in time and place and not necessarily the emergence of a new DENV-4 genotype in Southern Thailand. However, further investigation would be necessary in order to follow the DENV-4 phylogenetic dynamic.

In conclusion, our study describes, for the first time, the genetic diversity of all four serotypes of DENV in Southern Thailand, providing important insights into their distributions in the region. We demonstrated that DENV is hyperendemic in Southern Thailand, rendering coinfection between serotypes and genotypes possible. We observed the co-circulation of multiple genotypes within DENV-2 to DENV 4: DENV-2 Cosmopolitan genotype co-circulated with Asian-I; DENV-3 genotype I with genotype III; and DENV-4 genotype I was prevalent while genotype II-B was detected after decades of absence. The observed re-emergence of several genotypes suggests constant movement and introduction of DENV strains in Southern Thailand. Our study urges further *in vivo* characterization of the local mosquito population to assess the possible gain of *in vivo* advantage for the new genotypes in Southern Thailand. Gain in vector competence may partly explain the difference in genotype distribution between the North and the South of Thailand. Our data highlights the high incidence of DENV serotype and genotype co-circulation, particularly in urban area, such as the city of Hat Yai and the area along the Malaysian border. It would be informative to evaluate how this hyperendemicity influences disease severity.

## Supporting information

S1 Table Information on dengue positive study population. (PDF)

S2 Table Primers used in this study. (PDF)

S3 Table Dengue virus sequences used in present study. (PDF)
