# Supplementary material for: Phylogenetic analysis revealed the co-circulation of four dengue virus serotypes in Southern Thailand
Source: PLoS One. 2019 Aug 15;14(8):e0221179. doi: 10.1371/journal.pone.0221179 (PMC6695175; doi:10.1371/journal.pone.0221179)
Supplement: S1 Table — (PDF) [file pone.0221179.s001.pdf]

S1 table. Information on Dengue positive study population.

no	Name	Age*	Gender**	Location	Onset Fever	Collection	Fever Drops	NS1***	Serotype
1	DC1	13	M	HatYai/Songkla	04/09/2015	07/09/2015	09/09/2015	Pos	3
2	DC2	47	M	Takbai/ Narathiwat	05/09/2015	07/09/2015	09/09/2015	Pos	3
3	DC3	21	F	HatYai/Songkla	04/09/2015	09/09/2015	09/09/2015	Pos	3
4	DC4	19	F	HatYai/Songkla	07/09/2015	10/09/2015	11/09/2015	WP	2
5	DC5	23	F	Saba Yoi/Songkla	08/09/2015	10/09/2015	12/09/2015	Pos	4
6	DC6	13	F	HatYai/Songkla	07/09/2015	11/09/2015	12/09/2015	Pos	3
7	DC8	22	M	HatYai/Songkla	12/09/2015	15/09/2015	17/09/2015	Pos	4
8	DC9	27	F	Khuan Don/Songkhla	14/09/2015	18/09/2015	20/09/2015	Pos	2
9	DC10	13	F	HatYai/Songkla	17/09/2015	20/09/2015	22/09/2015	Pos	2
10	DC11	20	M	HatYai/Songkla	25/09/2015	28/09/2015	30/09/2015	Pos	2
11	DC12	54	F	HatYai/Songkla	27/09/2015	29/09/2015	02/10/2015	Pos	3
12	DC13	48	M	HatYai/Songkla	26/09/2015	29/09/2015	01/10/2015	WP	2
13	DC14	35	F	HatYai/Songkla	15/12/2015	18/12/2015	19/12/2015	Pos	2
14	DC15	21	M	HatYai/Songkla	31/12/2015	05/01/2016	06/01/2016	WP	2
15	DC16	16	M	HatYai/Songkla	06/01/2016	08/01/2016	10/01/2016	WP	2
16	DC17	38	F	HatYai/Songkla	26/08/2015	29/08/2015	02/09/2015	Pos	1
17	DC18	35	M	HatYai/Songkla	03/10/2015	05/10/2015	09/10/2015	Pos	4
18	DC19	23	M	HatYai/Songkla	01/10/2015	06/10/2015	06/10/2015	Pos	4
19	DC20	15	M	Bang Klam/Songkla	04/10/2015	07/10/2015	10/10/2015	Pos	2
20	DC21	5	M	HatYai/Songkla	09/10/2015	10/10/2015	13/10/2015	WP	2
21	DC22	6	M	HatYai/Songkla	08/10/2015	12/10/2015	13/10/2015	Pos	4
22	DC23	25	F	Pakpayoon/Songkhla	12/10/2015	13/10/2015	nd	Pos	2
23	DC24	11	M	HatYai/Songkla	12/10/2015	14/10/2015	15/10/2015	Pos	3
24	DC25	8	F	Bang Klam/Songkla	12/10/2015	15/10/2015	16/10/2015	Pos	2
25	DC26	33	F	HatYai/Songkla	14/10/2015	17/10/2015	18/10/2015	Pos	2
26	DC27	12	M	HatYai/Songkla	16/10/2015	19/10/2015	20/10/2015	Pos	3
27	DC28	15	M	HatYai/Songkla	24/10/2015	27/10/2015	29/10/2015	Pos	4
28	DC30	24	M	Bang Klam/Songkla	25/10/2015	28/10/2015	30/10/2015	Pos	2
29	DC31	8	F	HatYai/Songkla	25/10/2016	29/10/2015	30/10/2016	Pos	2
30	DC32	20	F	HatYai/Songkla	28/10/2015	30/10/2015	03/11/2015	Pos	3
31	DC33	2	F	Sadao/ Songkhla	31/10/2015	03/11/2015	nd	Pos	4
32	DC34	25	M	HatYai/Songkla	03/11/2015	40/11/2015	08/11/2015	Pos	2
33	DC35	10	F	Na Mon/Songkhla	02/11/2015	06/11/2015	06/11/2015	Pos	2
34	DC36	28	F	HatYai/Songkla	05/11/2015	08/11/2015	nd	Pos	4
35	DC37	28	F	HatYai/Songkla	10/11/2015	13/11/2015	nd	WP	4
36	DC38	20	F	HatYai/Songkla	15/11/2015	16/11/2015	20/11/2015	Pos	4
37	DC39	30	F	Khuan Kalong/Songkhla	18/11/2015	21/11/2015	23/11/2015	Pos	2
38	DC40	12	M	HatYai/Songkla	19/11/2015	22/11/2015	nd	Pos	2
39	DC41	28	M	HatYai/Songkla	23/11/2015	24/11/2015	30/11/2015	Pos	4

40	DC42	19	F	Rattaphum/Songkhla	23/11/2015	27/11/2015	27/11/2015	Pos	4
41	DC43	40	F	HatYai/Songkla	11/12/2015	14/12/2015	16/12/2015	Pos	2
42	DC44	20	F	HatYai/Songkla	15/12/2015	16/12/2015	19/12/2015	Pos	4
43	DC45	19	F	Bangkok/Bangkok	10/01/2016	13/01/2016	14/01/2016	Pos	4
44	DC46	30	F	HatYai/Songkla	10/01/2016	14/01/2016	nd	Pos	4
45	DC47	58	F	HatYai/Songkla	18/01/2016	22/01/2016	nd	Pos	2
46	DC49	27	M	Tung Song/ Nakhon Si Thammarat	10/02/2016	12/02/2016	nd	Pos	2
47	DC50	29	M	Takbai/ Narathiwat	10/02/2016	14/02/2016	nd	Pos	3
48	DC51	37	F	HatYai/Songkla	11/02/2016	14/02/2016	14/02/2016	Pos	2
49	DC52	18	F	Cha na/Songkla	16/02/2016	21/02/2016	nd	Pos	3
50	DC53	8	M	Pak Phanang/Nakhon Si Thammarat	16/03/2016	18/03/2016	22/03/2016	Pos	2
51	DC54	24	M	HatYai/Songkla	23/03/2016	24/03/2016	28/03/2016	Pos	4
52	DC55	17	F	HatYai/Songkla	21/03/2016	25/03/2016	26/03/2016	Pos	2
53	DC56	7	F	HatYai/Songkla	23/03/2016	27/03/2016	29/03/2016	Pos	4
54	DC57	13	M	Sadao/ Songkhla	08/04/2016	10/08/2559	12/04/2016	WP	2
55	DC58	12	F	Satun/Satun	16/04/2016	18/04/2559	21/04/2016	Pos	2
56	DC61	11	F	Khlong Hoi Khong/ Songkhla	10/05/2016	13/05/2016	13/05/2016	Pos	2
57	DC62	8	M	Khlong Hoi Khong/ Songkhla	13/05/2016	16/05/2016	17/05/2016	Pos	2
58	DC63	5	M	Sadao/ Songkhla	01/06/2016	06/06/2016	06/06/2016	Pos	2
59	DC64	23	M	Sadao/ Songkhla	07/06/2016	09/06/2016	11/06/2016	Pos	2
60	DC65	22	F	HatYai/Songkla	13/06/2016	15/06/2016	17/06/2016	Pos	2
61	DC66	28	M	HatYai/Songkla	13/06/2016	16/06/2016	19/06/2016	Pos	2
62	DC67	62	M	HatYai/Songkla	12/06/2016	16/06/2016	17/06/2016	Pos	4
63	DC68	31	M	HatYai/Songkla	01/08/2016	03/08/2016	06/08/2016	Pos	2
64	DC69	13	M	Sadao/ Songkhla	03/08/2016	05/08/2016	07/08/2016	Pos	4
65	DC70	20	F	HatYai/Songkla	06/08/2016	09/08/2016	09/08/2016	Pos	2
66	DC71	11	F	Songkhla/Songkla	04/08/2016	08/08/2016	09/08/2016	Pos	2
67	DC72	25	F	La-ngu/Satun	06/08/2016	09/08/2016	nd	Pos	2
68	DC73	8	F	Sadao/ Songkhla	13/08/2016	14/08/2016	nd	Pos	2
69	DC74	22	M	HatYai/Songkla	15/08/2016	16/08/2016	20/08/2016	Pos	2
70	DC77	55	F	Rattaphum/Songkhla	16/08/2016	19/08/2016	20/08/2016	Pos	2
71	DC78	23	F	HatYai/Songkla	21/08/2016	25/08/2016	26/08/2016	Pos	3
72	DC79	7	F	HatYai/Songkla	23/08/2016	26/08/2016	28/08/2016	Pos	2
73	DC80	15	M	HatYai/Songkla	08/09/2016	12/09/2016	13/09/2016	Pos	2
74	DC81	21	M	HatYai/Songkla	12/09/2016	13/09/2016	18/09/2016	Pos	2
75	DC82	14	M	HatYai/Songkla	10/09/2016	13/09/2016	14/09/2016	Pos	2
76	DC83	19	F	HatYai/Songkla	12/09/2016	14/09/2016	15/09/2016	Pos	4
77	DC85	13	F	HatYai/Songkla	15/09/2016	18/09/2016	18/09/2016	Pos	4
78	DC88	64	M	HatYai/Songkla	21/09/2016	24/09/2016	26/09/2016	Pos	2
79	DC89	60	M	Rattaphum/Songkhla	23/09/2016	25/09/2016	nd	Pos	2

80	DC90	9	F	Songkhla/Songkla	28/08/2016	31/08/2016	nd	Pos	2
81	DC91	27	F	HatYai/Songkla	01/09/2016	02/09/2016	05/09/2016	Pos	2
82	DC92	17	M	Songkhla/Songkla	03/09/2016	06/09/2016	08/09/2016	Pos	2
83	DC95	27	F	Songkhla/Songkla	25/09/2016	28/09/2016	01/10/2016	Pos	2
84	DC97	50	F	HatYai/Songkla	31/09/2016	03/10/2016	nd	Pos	2
85	DC98	18	F	Cha na/Songkla	03/10/2016	05/10/2016	06/10/2016	Pos	2
86	DC99	9	M	Rattaphum/Songkhla	04/10/2016	05/10/2016	06/10/2016	Pos	2
87	DC100	15	F	HatYai/Songkla	03/10/2016	06/10/2016	09/10/2016	Pos	4
88	DC101	7	F	HatYai/Songkla	08/10/2016	09/10/2016	11/10/2016	Pos	2
89	DC103	33	F	HatYai/Songkla	08/10/2016	10/10/2016	10/10/2016	Pos	2
90	DC104	46	F	Cha na/Songkla	06/10/2016	10/10/2016	nd	Pos	4
91	DC105	13	M	HatYai/Songkla	09/10/2016	12/10/2016	nd	Pos	2
92	DC106	18	M	HatYai/Songkla	10/10/2016	13/10/2016	13/10/2016	Pos	2
93	DC107	31	F	Sadao/ Songkhla	11/10/2016	15/10/2016	15/10/2016	Pos	2
94	DC108	26	F	HatYai/Songkla	12/10/2016	14/10/2016	17/10/2016	Pos	2
95	DC109	8	M	HatYai/Songkla	13/10/2016	16/10/2016	17/10/2016	Pos	2
96	DC110	27	M	Pak Phayun/Phattalung	14/10/2016	18/10/2016	nd	Pos	2
97	DC111	11	F	HatYai/Songkla	19/10/2016	21/10/2016	23/10/2016	Pos	2
98	DC112	12	F	HatYai/Songkla	20/10/2016	21/10/2016	25/10/2016	Pos	2
99	DC113	9	F	Satun/Satun	22/10/2016	25/10/2016	27/10/2016	Pos	4
100	DC114	68	M	Na Mon/Songkhla	22/10/2016	25/10/2016	27/10/2016	Pos	2
101	DC115	44	M	Songkhla/Songkla	21/10/2016	25/10/2016	25/10/2016	Pos	2
102	DC116	30	F	HatYai/Songkla	24/10/2016	27/10/2016	27/10/2016	Pos	2
103	DC117	28	F	Cha na/Songkla	26/10/2016	29/10/2016	30/10/2016	Pos	2
104	DC118	19	M	HatYai/Songkla	28/10/2016	31/10/2016	01/11/2016	Pos	2
105	DC119	18	F	HatYai/Songkla	29/10/2016	31/10/2016	nd	Pos	2
106	DC120	28	M	HatYai/Songkla	04/11/2016	04/11/2016	07/11/2016	Pos	2
107	DC121	16	F	Sathing Phra/Songkla	03/11/2016	04/11/2016	08/11/2016	Pos	3
108	DC122	29	M	Khlong Hoi Khong/ Songkhla	03/11/2016	05/11/2016	09/11/2016	Pos	3
109	DC123	9	F	Na Mon/Songkhla	03/11/2016	06/11/2016	09/11/2016	Pos	2
110	DC124	17	F	HatYai/Songkla	04/11/2016	07/11/2016	09/11/2016	Pos	2
111	DC125	29	M	HatYai/Songkla	05/11/2016	08/11/2016	10/12/2016	Pos	3
112	DC126	14	F	Rattaphum/Songkhla	06/11/2016	09/11/2016	nd	Pos	2
113	DC127	36	F	HatYai/Songkla	07/11/2016	07/11/2016	09/11/2016	Pos	2
114	DC130	22	F	HatYai/Songkla	08/12/2016	10/12/2016	13/12/2016	Pos	3
115	DC131	27	F	HatYai/Songkla	09/12/2016	10/12/2016	nd	Pos	3
116	DC132	26	M	Bang Klam/Songkla	08/12/2016	11/12/2016	nd	Pos	2
117	DC133	34	F	HatYai/Songkla	09/12/2016	11/12/2016	15/12/2016	Pos	3
118	DC134	19	M	HatYai/Songkla	09/12/2016	11/12/2016	13/12/2016	Pos	2
119	DC136	15	M	HatYai/Songkla	08/12/2016	12/12/2016	14/12/2016	Pos	2
120	DC137	7	M	Sadao/ Songkhla	09/12/2016	13/12/2016	14/12/2016	Pos	3

121	DC138	20	F	Khlong Hoi Khong/ Songkhla	14/12/2016	15/12/2016	17/12/2016	Pos	4
122	DC139	20	F	HatYai/Songkla	14/12/2016	17/12/2016	nd	Pos	2
123	DC140	11	M	HatYai/Songkla	15/12/2016	18/12/2016	nd	Pos	3
124	DC141	7	M	HatYai/Songkla	17/12/2016	18/12/2016	22/12/2016	Pos	2
125	DC142	18	F	HatYai/Songkla	17/12/2016	20/12/2016	22/12/2016	Pos	2
126	DC143	27	F	Sadao/ Songkhla	20/12/2016	21/12/2016	25/12/2016	Pos	2
127	DC144	18	M	HatYai/Songkla	18/12/2016	21/12/2016	24/12/2016	Pos	2
128	DC145	25	F	HatYai/Songkla	21/12/2016	24/12/2016	26/12/2016	Pos	2
129	DC146	40	F	Rattaphum/Songkhla	25/12/2016	29/12/2016	nd	Pos	2
130	DC147	23	F	HatYai/Songkla	29/12/2016	31/12/2016	nd	Pos	2
131	DC148	50	F	HatYai/Songkla	31/12/2016	02/1/2017	nd	Pos	2
132	DC149	35	F	HatYai/Songkla	31/12/2016	04/1/2017	07/1/2017	Pos	2
133	DC150	38	F	Khlong Hoi Khong/ Songkhla	01/1/2017	04/1/2017	06/1/2017	Pos	2
134	DC151	20	F	Khlong Hoi Khong/ Songkhla	01/1/2017	04/1/2017	05/1/2017	Pos	2
135	DC152	12	M	HatYai/Songkla	03/1/2017	05/1/2017	08/1/2017	Pos	2
136	DC153	17	F	HatYai/Songkla	04/1/2017	07/1/2017	08/1/2017	Pos	2
137	DC154	22	F	HatYai/Songkla	07/1/2017	09/1/2017	12/1/2017	Pos	2
138	DC155	39	F	HatYai/Songkla	06/1/2017	09/1/2017	nd	Pos	2
139	DC157	19	F	HatYai/Songkla	10/1/2017	12/1/2017	15/1/2017	Pos	2
140	DC159	23	F	HatYai/Songkla	17/1/2017	20/1/2017	22/1/2017	Pos	4
141	DC160	37	F	HatYai/Songkla	16/1/2017	20/1/2017	22/1/2017	Pos	2
142	DC162	12	F	Na Mon/Songkhla	20/1/2017	21/1/2017	25/1/2017	Pos	2
143	DC163	7	F	HatYai/Songkla	19/1/2017	22/1/2017	25/1/2017	Pos	2
144	DC164	13	M	HatYai/Songkla	21/12017	22/1/2017	25/1/2017	Pos	2
145	DC165	22	F	HatYai/Songkla	23/1/2017	25/1/2017	30/1/2017	Pos	2
146	DC166	14	M	Sadao/ Songkhla	22/1/2017	25/1/2017	nd	Pos	2
147	DC167	19	F	HatYai/Songkla	21/1/2017	25/1/2017	26/1/2017	Pos	2
148	DC168	15	M	HatYai/Songkla	24/1/2017	27/1/2017	30/1/2017	Pos	2
149	DC169	45	M	HatYai/Songkla	29/1/2017	30/1/2017	04/2/2017	Pos	4
150	DC170	18	M	Songkhla/Songkla	27/1/2017	30/1/2017	nd	Pos	2
151	DC172	21	F	HatYai/Songkla	31/1/2017	02/2/2017	05/2/2017	Pos	3
152	DC174	25	F	HatYai/Songkla	02/2/2017	05/2/2017	08/2/2017	Pos	2
153	DC175	38	M	Khlong Hoi Khong/ Songkhla	03/2/2017	05/2/2017	07/2/2017	Pos	2
154	DC176	22	F	HatYai/Songkla	01/2/2017	05/2/2017	06/2/2017	Pos	2
155	DC177	12	F	HatYai/Songkla	06/2/2017	08/2/2017	10/2/2017	Pos	2
156	DC185	68	M	HatYai/Songkla	24/3/2017	25/3/2017	27/3/2017	Pos	3
157	DC186	27	F	Rattaphum/Songkhla	26/3/2017	28/3/2017	31/3/2017	Pos	3
158	DC187	13	M	HatYai/Songkla	31/3/2017	02/4/2017	06/3/2017	Pos	2
159	DC188	12	M	HatYai/Songkla	01/4/2017	05/4/2017	06/3/2017	Pos	2
160	DC189	38	F	HatYai/Songkla	22/12/2016	23/12/2016	26/12/2016	WP	2

161	DC190	36	M	HatYai/Songkla	30/12/2016	30/12/2016	nd	WP	2
162	DC191	15	F	Songkhla/Songkla	21/12017	26/1/2017	nd	WP	2
163	DC193	8	F	HatYai/Songkla	01/2/2017	02/2/2017	06/2/2017	WP	2
164	DC194	4	F	HatYai/Songkla	05/2/2017	09/2/2017	12/2/2017	WP	2
165	DC195	53	F	Songkhla/Songkla	12/2/2017	13/2/2017	nd	WP	4
166	DC196	22	M	HatYai/Songkla	15/2/2017	17/2/2017	21/2/2017	WP	4
167	DC197	34	F	HatYai/Songkla	01/3/2017	02/3/2017	05/2/2017	WP	2
168	DC198	42	M	HatYai/Songkla	29/4/2017	01/4/2017	02/4/2017	WP	2
169	DC199	34	F	Na Mon/Songkhla	29/3/2017	02/4/2017	nd	WP	2
170	DC200	39	F	HatYai/Songkla	28/10/2016	1/11/2016	02/11/2016	Pos	2
171	DC201	15	F	Sadao/ Songkhla	14/11/2016	15/11/2016	nd	Pos	1
172	DC202	19	M	Rattaphum/Songkhla	15/11/2016	17/11/2016	nd	Pos	1
173	DC203	16	F	HatYai/Songkla	16/11/2016	17/11/2016	21/11/2016	Pos	2
174	DC204	34	F	HatYai/Songkla	16/11/2016	18/11/2016	20/11/2016	Pos	4
175	DC205	26	M	HatYai/Songkla	14/11/2016	19/11/2016	20/11/2016	Pos	2
176	DC206	21	M	Sadao/ Songkhla	18/11/2016	19/11/2016	22/11/2016	Pos	2
177	DC207	18	M	Songkhla/Songkla	01/12/2016	02/12/2016	07/11/2016	Pos	2
178	DC211	22	F	Bang Klam/Songkla	20/11/2016	23/11/2016	nd	Pos	2
179	DC213	34	M	HatYai/Songkla	20/11/2016	23/11/2016	27/11/2016	Pos	2
180	DC214	34	F	Rattaphum/Songkhla	23/11/2016	25/11/2016	29/11/2016	Pos	3
181	DC216	9	M	HatYai/Songkla	25/11/2016	26/11/2016	29/11/2016	Pos	4
182	DC218	12	F	HatYai/Songkla	26/11/2016	27/11/2016	30/11/2016	Pos	4

Origins of dengue strains used in the study

*Age of patients in years.
**F: female, M: male
*** WP : weak positive; Pos: positive
