# Supplementary material for: Phylogenetic analysis revealed the co-circulation of four dengue virus serotypes in Southern Thailand
Source: PLoS One. 2019 Aug 15;14(8):e0221179. doi: 10.1371/journal.pone.0221179 (PMC6695175; doi:10.1371/journal.pone.0221179)
Supplement: S2 Table — (PDF) [file pone.0221179.s002.pdf]

S2 table. Primers used in this study

Primers			Sequences (5'-3')	Amplicon size (pb)
For Nested-PCR				
D1F	1st and 2nd PCR	Forward	5-TCAATATGCTGAAACGCGCGAGAAACCG-3	656
D1R	1st PCR	Reverse	5-CAAAGCYCCTTCMGMYGACAT-3	
RTS1	2nd PCR	Reverse	5-CGTCTCAGTGATCCGGGGRG-3	482
RTS2	2nd PCR	Reverse	5-CGCCACAAGGGCCATGAACAG-3	119
RTS3	2nd PCR	Reverse	5-TAACATCATCATGAGACAGAGC-3	290
RTS4	2nd PCR	Reverse	5-CTCTGTTGTCTTAAACAAGAGA-3	392
For sequencing				
Envelope DENV-1	1st	Forward	5-ATGCGATGCGTGGGAATAGG-3	438
		Reverse	5-TCCAGTGTGGACGGTGACTA-3	
	2nd	Forward	5-AAAACTGGAAGGAAAAATAGTTCAA-3	453
		Reverse	5-GACAGAAATCCAAACGTCTGG-3	
	3rd	Forward	5-CATGCAAAGAAGCAGGAAGT-3	460
		Reverse	5-TGCTTCCTTTCTTGAACCAG-3	
	4th	Forward	5-CTGGTTCAAGAAAGGAAGCA-3	316
		Reverse	5-CGCCTGAACCATGACTCCTA-3	
Envelope DENV-2	1st	Forward	5-TGCGTTGCATAGGAATATCA-3	447
		Reverse	5-CATGCTCCTCCCCTGAGT-3	
	2nd	Forward	5-GTGCAACCAGAAAACTTGGA-3	477
		Reverse	5-TCTCAGCCTGCACTTGAGAT-3	
	3rd	Forward	5-TGCATACAGCACTCACAGGA-3	430
		Reverse	5-TCAAACATTTGGCCGATAGA-3	
	4th	Forward	5-AGTAGAGCCGGGACAACTGA-3	343
		Reverse	5-GGCCTGCACCATGACTC-3	
Envelope DENV-3	1st	Forward	5-ATGAGATGTGTGGGAGTAGGAAAC-3	450
		Reverse	5-CTGGTGTTGGTCTCCTGTGT-3	
	2nd	Forward	5-AGGGAAAAGTGGTGCAATATG-3	450
		Reverse	5-TGCTCGTACCTCCTGAATTTT-3	
	3rd	Forward	5-GAGGGAGCAATGCACACA-3	423
		Reverse	5-CGAGCTTCCCTTCTTGTACC-3	
	4th	Forward	5-TGGGGAAAGCAACATAGTGA-3	367
		Reverse	5-AGCTTGTACCACGGCTCC-3	
Envelope DENV-4	1st	Forward	5-ATGCGATGCGTAGGAGTAGG-3	450
		Reverse	5-AGCGTGAGTGTCTCCATTGT-3	
	2nd	Forward	5-AGTCCAAATTGAGAACCTTGAA-3	436
		Reverse	5-ATCACCGGAATCCACTTCTG-3	
	3rd	Forward	5-GAAGGAGCCATGCATTCAG-3	414
		Reverse	5-TTTCCTGAACCAATGGAGTG-3	
	4th	Forward	5-TCCTTTCGGGGATAGCTACA-3	373
		Reverse	5-TGCATGGACTGTGAAGCC-3	
