# Supplementary material for: Phylogenetic analysis revealed the co-circulation of four dengue virus serotypes in Southern Thailand
Source: PLoS One. 2019 Aug 15;14(8):e0221179. doi: 10.1371/journal.pone.0221179 (PMC6695175; doi:10.1371/journal.pone.0221179)
Supplement: S3 Table — (PDF) [file pone.0221179.s003.pdf]

S3 table. Dengue virus sequences used in present study.

Serotype	Genotype	Accession number	Country	Year
DENV-1	I	JN415529	Australia	2008
		KF955406	Cambodia	2001
		AB873104	Cambodia	2013
		KJ470729	China	2013
		KT232191	China	2014
		LC148029	Indonesia	2007
		KT175091	Indonesia	2014
		KT175093	Indonesia	2014
		AB074760	Japan	1943
		LC002828	Japan	2014
		KC182107	Laos	2008
		KC182091	Laos	2010
		KJ806873	Malaysia	2014
		KT825001	Myanmar	2013
		EU081266	Singapore	2005
		KJ806960	Singapore	2014
		JN415529	Southeast Asia	2005
		JQ403519	Taiwan	2009
		AY732477	Thailand	1991
		JN415525	Thailand	2001
		FJ850068	Thailand	2001
		JQ993141	Thailand	2007
		JQ993151	Thailand	2007
		KT373902	Thailand	2009
		KT824981	Thailand	2009
		JN415528	Thailand	2010
		KY882504	Thailand	2013
		KF887994	Thailand	2013
		KY495792	Thailand	2015
		LC410183	Thailand	2017
		JF967859	Vietnam	2009
		KY818252	Vietnam	2012
	II	AF425629	Thailand	1963
	III	EF457905	Malaysia	1972
		AF425622	Malaysia	1972
	IV	AB074761	Indonesia	1988
		KC762651	Indonesia	2007
		DQ285560	La Reunion	2004
		JN415513	Malaysia	2010
		U88535	Nauru Island	1974
		LC148027	Philippines	2012
		KY818205	Philippines	2003
	V	AF425613	Brazil	1982
		KY818289	Brazil	1982
		AF425620	Ivory Cost	1985
		AF425621	Jamaica	1977
		AY722803	Myanmar	1998

DENV-2	Asian I	DQ518649	Cambodia	2003
		JF967986	Cambodia	2009
		KY038915	China	2014
		KX262942	China	2015
		KX262927	China	2015
		JN568244	Laos	2010
		LC147056	Laos	2013
		DQ518651	Myanmar	2002
		KJ470760	Myanmar	2013
		KX357994	Myanmar	2015
		KT175111	Taiwan	2011
		NC001474	Thailand	1964
		AF195036	Thailand	1994
		GQ868545	Thailand	1996
		GU131886	Thailand	2001
		JF812111	Thailand	2006
		JQ993211	Thailand	2007
		JN568273	Thailand	2007
		JN568274	Thailand	2010
		KT175135	Thailand	2014
		LC410184	Thailand	2016
		JN568282	Vietnam	2010
	Asian II	FJ906959	Papua New Guinea	2008
		L10045	Philippines	1983
		AF295697	Philippines	2000
	Asia-America	GQ368167	Brazil	2008
		KX901652	Colombia	2015
		KM279422	Mexico	2012
		AF195040	Thailand	1991
		GU211755	Vietnam	2006
	America	AF100467	Peru	1999
		DQ341198	Puerto Rico	1977
	Cosmopolitan	KT781521	Australia	2010
		JN036373	Bangladesh	2009
		KY038897	China	2015
		EU005258	Ghana	2005
		JN568259	India	2010
		JN568247	Indonesia	2009
		JN568272	Indonesia	2010
		KY709184	Indonesia	2015
		KR779785	Indonesia	2015
		KT716076	Kenya	2013
		JF967962	Malaysia	2008
		KJ806875	Malaysia	2012
		KJ806876	Malaysia	2012
		KJ806778	Malaysia	2013
		KJ806895	Malaysia	2013
		KX452017	Malaysia	2014
		KT806325	Malaysia	2014
		KT781520	Papua New Guinea	2010

		JN568264	Philippines	2010
		LC410190	Thailand	2016
		LC410189	Thailand	2016
		KY275213	Timor	2011
		KR779785	Singapore	2013
		KX224268	Singapore	2014
		M24449	Sri Lanka	1989
		KY495803	Sri Lanka	2016
DENV-3	I	JN575579	Australia	2009
		JN029823	China	2010
		L11426	Indonesia	1978
		AY858045	Indonesia	2004
		KC762686	Indonesia	2007
		KC762693	Indonesia	2010
		KM216738	Indonesia	2010
		FM986662	Malaysia	1997
		EU448438	Malaysia	2007
		JF968112	Malaysia	2010
		EU182246	New Caledonia	1989
		AY496879	Philippines	1997
		KJ946243	Philippines	2008
		DQ518672	Taiwan	2005
		AY145714	Thailand	1988
		DQ453979	Timor	2005
		JN030164	Singapore	2009
	II	JN575565	Cambodia	2006
		HG530147	Laos	2012
		HG530202	Laos	2013
		EU448444	Myanmar	2006
		JF968066	Myanmar	2008
		JN030195	Singapore	2009
		GQ868593	Thailand	1973
		DQ863638	Thailand	1973
		L11620	Thailand	1973
		L11441	Thailand	1986
		AY923865	Thailand	1994
		AY676365	Thailand	1996
		JN575574	Thailand	1997
		FJ687448	Thailand	2001
		FJ744739	Thailand	2001
		JQ993229	Thailand	2005
		JF812105	Thailand	2006
		JF968093	Thailand	2010
		JN575576	Thailand	2010
		KT758742	Thailand	2011
		KT758791	Thailand	2013
		HQ588145	Vietnam	2006
		JN575580	Vietnam	2008
	III	DQ118882	Brazil	2004
		AB111081	Cambodia	2000

		KT758770	Cambodia	2012
		KT187285	Cape Verde	2009
		KU053472	French Polynesia	2013
		JQ686079	India	2010
		KT187283	Ivory Coast	2008
		AY099337	La Martinique	1999
		KT187288	La Reunion	2012
		KF816163	Laos	2013
		KT187291	Madagascar	2010
		JF968068	Malaysia	2008
		KX380842	Singapore	2013
		JF968098	Thailand	2010
		KP176707	Thailand	2012
		KP176708	Thailand	2013
		KY851618	Thailand	2013
		KY495823	Thailand	2015
		KT758784	Thailand	2015
		LC410193	Thailand	2016
		LC410195	Thailand	2017
		KP176710	Vietnam	2013
		KP893717	Vietnam	2013
	V	KU050695	Philippines	1956
	VI	AY146762	Puerto Rico	1963
DENV-4	I	JN638571	Cambodia	2007
		KF543273	Cambodia	2011
		MG601754	China	2013
		KX262926	China	2015
		KJ938507	India	2012
		KY427081	Indonesia	2016
		KY849762	Lao	2009
		EU478408	Myanmar	2006
		KR051897	Myanmar	2013
		KX224312	Singapore	2014
		KY427080	Sri Lanka	2016
		AY618991	Thailand	1977
		AB111087	Thailand	1999
		AY618977	Thailand	1996
		EU448457	Thailand	2003
		JQ993306	Thailand	2006
		JQ993252	Thailand	2007
		KR922405	Thailand	2011
		KT749998	Thailand	2011
		KT026308	Thailand	2011
		KY451945	Thailand	2013
		KY234182	Thailand	2013
		LC410201	Thailand	2016
		LC410199	Thailand	2016
		LC410197	Thailand	2016
		LC410196	Thailand	2016
		LC410202	Thailand	2017

		EU448450	Vietnam	2006
		KP292922	Vietnam	2013
	II-A	KP704217	Brazil	2013
		KX901660	Colombia	2014
		KU728215	Dominica	2006
		FM986664	Malaysia	1999
		JF804055	Mexico	2006
		FJ850059	Puerto Rico	1998
		KF809762	Puerto Rico	2013
	II-B	JX878504	Australia	2012
		JN832555	French Polynesia	2010
		JF967782	Indonesia	2010
		KM216747	Indonesia	2011
		KU529756	Indonesia	2014
		KY709196	Indonesia	2015
		FM986674	Malaysia	2002
		JN575590	Malaysia	2009
		KY851684	Malaysia	2012
		KY427082	Papua New Guinea	2016
		JN019816	Singapore	2010
		KX224302	Singapore	2013
		JQ403526	Taiwan	2010
		AY618993	Thailand	2000
		KY275254	Timor	2010
	III	AY618988	Thailand	1997
		KY586945	Thailand	1998
